# A Self-Guided Lifestyle Intervention for Young Men: Findings from the ACTIVATE Randomized Pilot Trial

**DOI:** 10.31083/j.jomh1809191

**Published:** 2022-09-14

**Authors:** Jean M. Reading, Melissa M. Crane, Kellie Carlyle, Robert A. Perera, Jessica Gokee LaRose

**Affiliations:** 1 Department of Health Behavior and Policy, Virginia Commonwealth University School of Medicine, Richmond, VA 23219, USA; 2 Department of Preventive Medicine, Rush University Medical Center, Chicago, IL 60612, USA; 3 Department of Biostatistics, Virginia Commonwealth University School of Medicine, Richmond, VA 23219, USA

**Keywords:** behavioral interventions, male-targeted, weight loss, health risk messages, low intensity, obesity

## Abstract

**Background:**

Young men are at high risk for developing obesity-related health complications, yet are markedly underrepresented in lifestyle interventions. This pilot study examined the feasibility and preliminary efficacy of a lifestyle intervention (self-guided + health risk messaging) targeting young men.

**Methods:**

35 young men (Age = 29.3 ± 4.27; BMI = 30.8 ± 4.26; 34% racial/ethnic minority) were randomly assigned to the intervention or delayed treatment control. The intervention (ACTIVATE) included 1 virtual group session, digital tools (wireless scale, self-monitoring app), access to self-paced content via a secure website, and 12 weekly texts to reinforce health risk messaging. Fasted objective weight was assessed remotely at baseline and 12-weeks. Perceived risk was assessed via survey at baseline, 2-week, and 12-week. *T*-tests were used to compare weight outcomes between arms. Linear regressions examined the association between percent weight change and perceived risk change.

**Results:**

Recruitment was successful as evidenced by 109% of target enrollment achieved in a 2-month period. Retention was 86% at 12 weeks, with no differences by arm (*p* = 0.17). Participants in the intervention arm experienced modest weight loss at 12 weeks, whereas slight gains were observed in the control arm (−1.6% ± 2.5 *vs.* +0.31% ± 2.8, *p* = 0.04). Change in perceived risk was not associated with change in percent weight (*p* > 0.05).

**Conclusions:**

A self-guided lifestyle intervention showed initial promise for weight management among young men, but these findings are limited by small sample size. More research is needed to bolster weight loss outcomes while retaining the scalable self-guided approach.

**Clinical Trial Registration:**

NCT04267263 (https://www.clinicaltrials.gov/ct2/show/NCT04267263).

## 1. Introduction

Nearly 40% of men between 20–39 years of age meet criteria for obesity (Body Mass Index (BMI) >30 kg/m^2^), with the prevalence increasing to over 46% after the age of 40 [1]. This is concerning given the links between obesity and cardiovascular disease [2] and cancer [3]—the two leading causes of death among men [4]. Young men in particular engage in a number of obesity-related health risk behaviors including high consumption of processed meats and alcohol [5] and low consumption of fruits and vegetables [6]. Considering the risk of obesity among men, it is imperative to promote weight loss during young adulthood (age 18–35). However, despite the prevalence of obesity among this population, young men are underrepresented in weight management trials—even those adapted specifically for young adults [7,8]. Therefore, more work is needed to close these gaps in enrollment and improve outcomes for men during this critical window in the developmental life course.

The challenge of enrolling men of all ages into lifestyle interventions has led to a burgeoning area of research testing the effects of male-only lifestyle interventions [9–12]. Formative data supporting male-only interventions indicate that men are more open to share weight loss experiences with other men and are more comfortable in programs designed specifically for them [13]. In addition, extant evidence suggests that the intensity and delivery mode of gold standard weight management programs might not be appealing to young men [8,14]. A recent systematic review by the US Preventive Services Task Force found that about 95% of behavioral counseling programs, addressing diet and physical activity to reduce cardiometabolic risk, involve either a high- (>360-minutes) or medium-level (3- to 360-minutes) of contact from a behavioral counselor [15]. Indeed, this level of intensity is at odds with time and convenience barriers reported by young men [16]. A lower intensity, male-only intervention might be more appealing to young men and limited available data suggest that men perform well in weight management programs that are self-guided (e.g., provide evidence-based content with none to minimal support) [8,17,18]. Yet, to our knowledge, this type of approach has not been designed for or tested among young men specifically. Yet, young men remain underrepresented in male-only lifestyle interventions [10,11]. As such, there remains a need for lifestyle interventions designed to meet both gender preferences and developmental needs of young men.

Given young men have low concern about weight [19,20], employing theory-based messaging within a self-guided lifestyle intervention, to raise health concerns associated with obesity, might be a natural fit. Health risk messages have played a key role in public health campaigns to alter health behaviors and increase perception of health risks [21,22]. One specific theory used to develop health risk messages is the extended parallel process model (EPPM). The EPPM suggests that our perception of a health threat interacts with the belief that a recommended behavior change strategy is effective at preventing the given health threat. When coupled with the confidence to carry out the given behavior, this will in turn, influence our intent to adopt the recommended behavior [23]. EPPM has been applied to a number of different health areas including smoking cessation [24] and breast cancer screening [22]. Nevertheless, obesity-related research with EPPM is minimal—though, EPPM shows some promise for changing risky health behaviors among young men. Limited evidence indicates that EPPM-based messaging can motivate young men to engage in physical activity and enhance their perceived risk of obesity [25,26]. Despite these promising findings, EPPM has not yet been applied to a male-targeted self-guided lifestyle intervention. EPPM might be a particularly appropriate framework to integrate into a lifestyle intervention designed for young men, given evidence that shows young men in particular have lower concern about weight gain relative to young women [19].

It is plausible that an intervention that is not only male-targeted, but also self-guided and grounded within the EPPM framework, could be a viable approach to promoting weight loss among young men. Not only could using this type of programming improve enrollment among this high-risk population, it also has the potential to promote awareness of health risks associated with obesity, as well as equip young men with the necessary skills to effectively manage their weight throughout the life course. To that end, the primary aim of the present study is to test the feasibility and preliminary efficacy of a male-targeted lifestyle intervention (self-guided intervention + EPPM) on weight change among young men. We will assess feasibility via retention and satisfaction data, and anticipate that young men in the intervention arm will manifest greater reductions in weight at 12-weeks compared to the delayed treatment control arm. We also aim to explore potential changes in perceived risk in response to the intervention and whether change in perceived risk is associated with weight loss.

## 2. Methods

### 2.1 Sample

Participants were eligible if they were between 18 and 35 years of age, with a body mass index (BMI) between 25–45 kg/m^2^. Exclusion criteria were selected primarily for safety reasons and included: an uncontrolled medical condition that might make it unsafe to engage in exercise without medical supervision, diagnosis of type 1 or type 2 diabetes, report of a heart condition or chest pain during rest, history of anorexia or bulimia nervosa, report of compensatory behaviors within last 3 months, or hospitalization for psychiatric condition in the last 12 months. Additional exclusion criteria included potential confounds or reasons that would prevent participants from being able to benefit from the treatment: participation in another program promoting weight loss, loss of ≥5% body weight within the last 3 months, not able to read/speak English, does not possess a mobile device or unwilling to receive text messages, or lives/resides outside of North America.

### 2.2 Recruitment and Screening

**Fig. 1.**
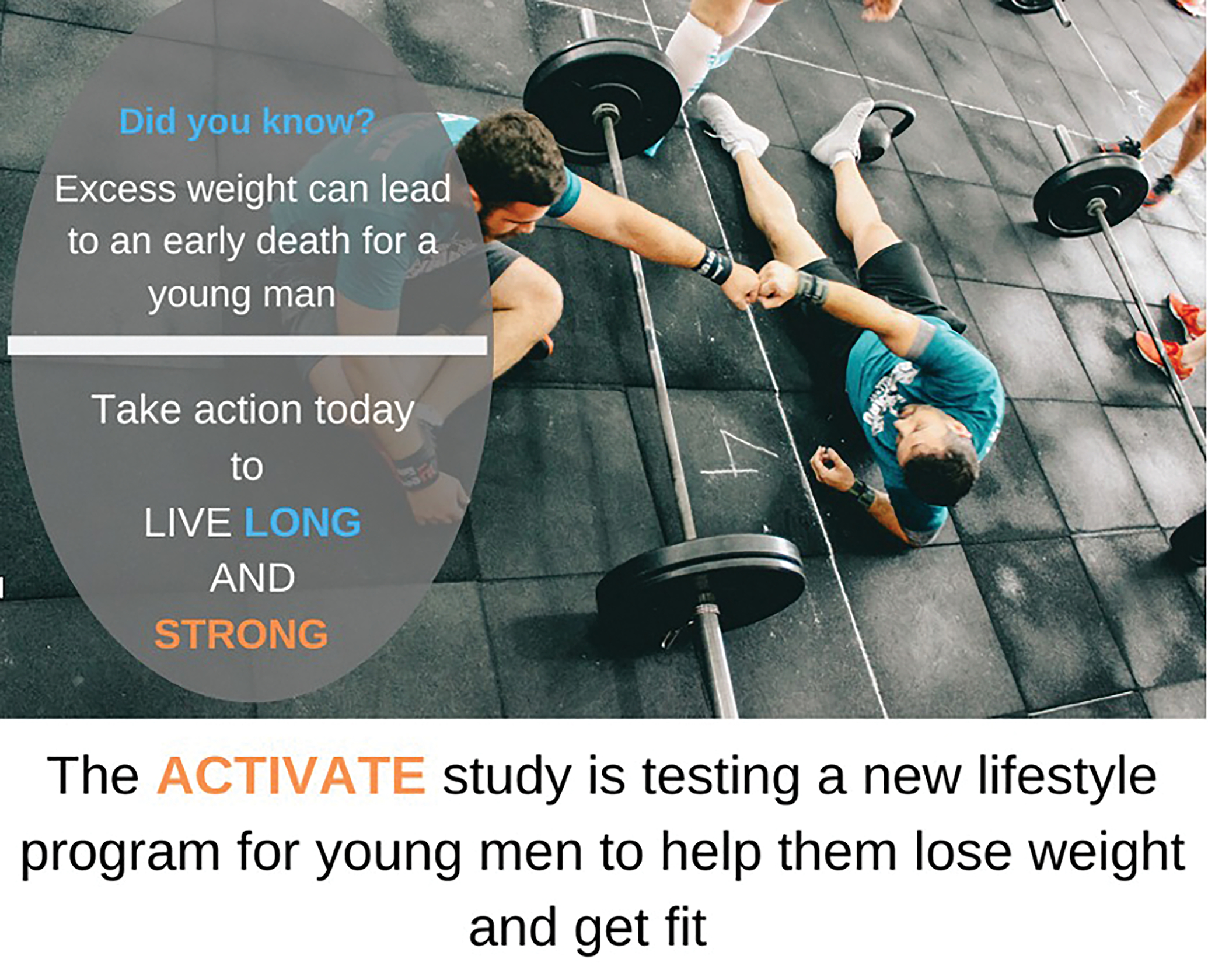
Example of male-target recruitment advertisement based in extended parallel process model.

Recruitment and screening occurred between January–March 2021 throughout North America. Both national and local recruitment strategies were adopted and included unpaid advertisements through Virginia Commonwealth University listservs, unpaid posts on social media, flyers distributed in university buildings and residence halls, and postings to research recruitment sites (e.g., https://www.researchmatch.org). Recruitment materials included an image of a young man engaging in physical activity paired with a male-targeted health risk message based in the EPPM. Messaging emphasized the risk of heart disease among men and that the program was a self-guided lifestyle program (See Fig. 1). A link to a recruitment website was included on all recruitment materials. The recruitment website provided interested individuals with a brief overview of the study, inclusion criteria, a BMI calculator, contact information, and a link to a secure online eligibility screener. Based on screening information, individuals who appeared eligible were contacted to attend a one-on-one virtual orientation session via Zoom. The interactive orientation session included a brief PowerPoint presentation with text/visuals and covered the study purpose and goals, study procedures, and time commitment. Time was also allotted for questions throughout and at the end. Following the brief presentation, those who remained interested began the informed consent process and were given the opportunity to review the consent form and ask questions. Participants who chose to enroll signed an informed consent form electronically.

### 2.3 Design

Eligible participants were stratified by BMI (25–35 kg/m^2^ or 36–45 kg/m^2^) and randomly assigned to 1 of 2 groups (ACTIVATE intervention or delayed treatment control). Blocks of four were used for the allocation sequence, which was generated by online software designed for this purpose and uploaded to REDCap. A trained research assistant (no role in either intervention or assessments) assigned and notified participants of assignment via phone. The trial protocol was approved by the institutional review board at Virginia Commonwealth University. The study had a target sample size of 32. The purpose was not to be a fully powered trial, but rather to obtain stable estimates of standard deviations and to determine if this self-guided model was a feasible approach to promote weight loss. The study had 80% power to detect a 3% between group comparison of change in weight from baseline to 12 weeks. All investigators and assessors were blinded until after the final data collection visit for this trial.

All protocol changes are reported using the CONSERVE Guidelines for reporting trial protocols (CONSERVE-SPIRIT Extension) [27]. The original in-person protocol was modified in May 2020, prior to the enrollment of the first participant, due to the global COVID-19 pandemic. Mitigating strategies included modifications that allowed for remote recruitment, data collection, intervention implementation, and measurement. Recruitment was expanded to all of North America given that in-person outcome assessments were no longer required. The protocol was revised to only examine weight change, as opposed to multiple measures of adiposity, given weight could be measured via a remote protocol that aligned with our clinic-based protocol (i.e., fasted state, serial objective measures on a study issued scale). Scale selection was based on data collected from reliability and validity testing of 4 different scales (Renpho, Taylor, Healthometer, Withings). A 15-pound hand weight was used during testing. The hand weight was placed on the scale 3 consecutive times and the weight was recorded. Measurements were also collected across 3 separate time points (approximately 1 week between each time point). Error for each scale was compared to a research grade clinic scale (Tanita BWB 800) and 3 other similar bathroom scales, and across time. The Renpho scale had 0 lb error within timepoint measurements. The error across timepoints was 0.2 lb, which was the same compared to all scales except the Taylor (0 error). The Renpho scale was selected based on cost, availability, and its Bluetooth capability. The intervention group kick-off session was adapted for delivery via Zoom instead of in-person.

Assessment visits occurred via Zoom (baseline and 12-weeks) and were identical to how assessments occur in clinic (e.g., fasted conditions, serial measures). Remote assessment visits were conducted by a blinded assessor in a private room. Video was only required to be on for the assessment of weight, in order for the assessor to see the weight shown on the scale. Participants were given the option to turn video off for the remaining measures.

### 2.4 Intervention

All participants received the 12-week ACTIVATE intervention that included 1 group kick-off session delivered via Zoom, access to a private intervention website with self-paced content, wireless/Bluetooth capable scale, 12 weekly text messages, and feedback reports at baseline and 12-weeks. Intervention content, text messages, and feedback reports included health risk messaging based on the 4 constructs of EPPM. Perceived threat is defined as perceived susceptibility (belief one is vulnerable to a specific disease) and perceived severity (belief the disease is serious). Efficacy is defined as self-efficacy (confidence to carry out recommendations to avoid risk of disease) and response efficacy (belief the proposed recommendation is effective at mitigating the disease risk) [23]. Health risk messaging emphasized the link between obesity and cardiovascular disease specific to young men, as well as the research evidence for the behavioral strategies taught to promote weight loss, and for weight loss to mitigate cardiovascular disease risk.

**Fig. 2.**
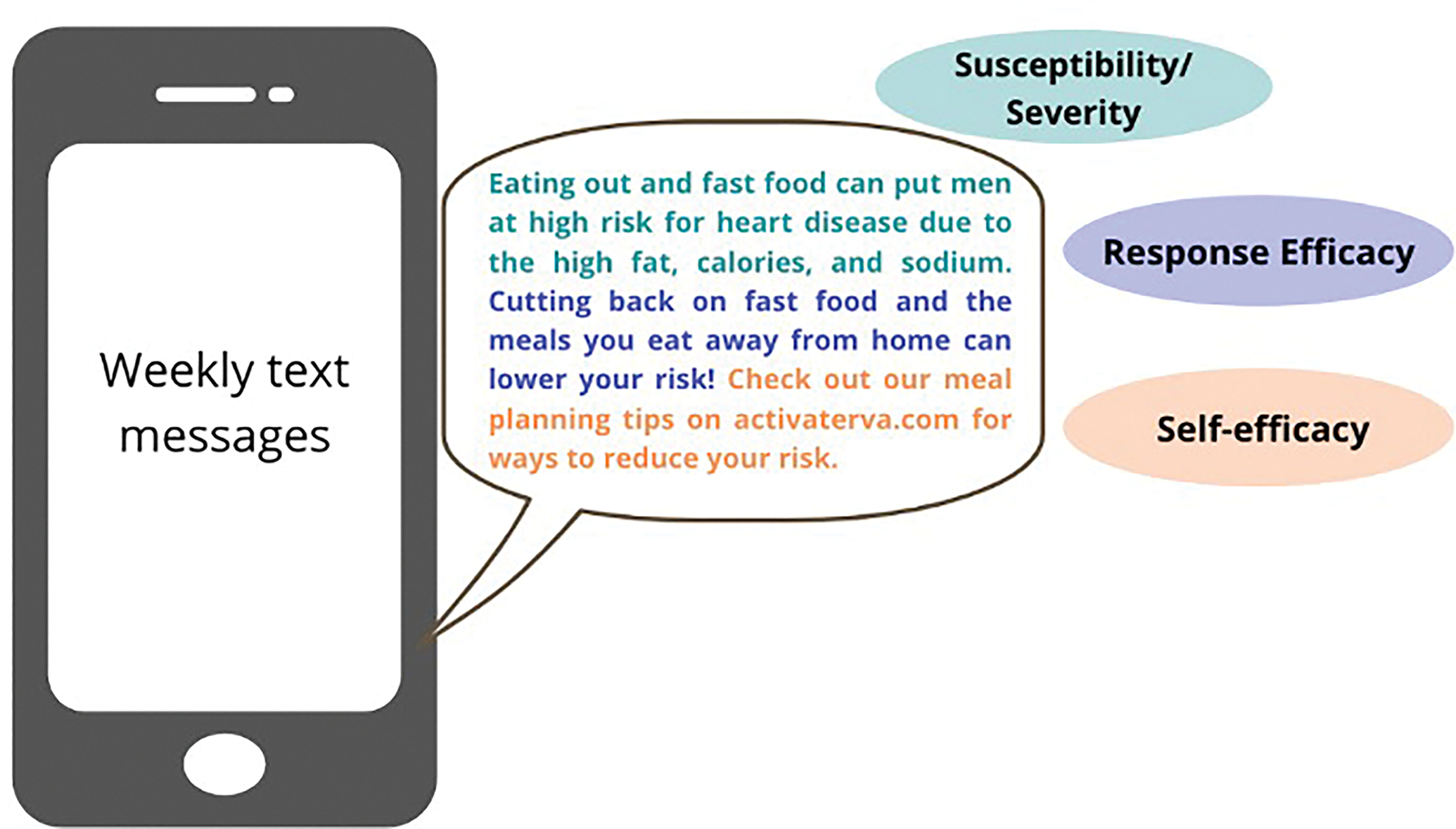
Example of weekly text message based in extended parallel process model.

The virtual group session was facilitated by a licensed clinical psychologist with expertise in behavioral weight loss treatment in young adults. The session provided an overview of health risks and a brief review of the principles of behavioral self-regulation for weight management [28]. During the session, participants received psychoeducation, training in evidence-based behavior change techniques, and engaged in skills practice to enhance self-efficacy. The intervention website provided participants with additional psychoeducation about healthy weight management practices including diet and meal prep strategies and physical activity recommendations. The website also provided content focused on evidence-based behavior change techniques associated with weight loss, including self-monitoring and goal setting [29,30]. All intervention content was adapted to enhance relevance and meet the needs and preferences of young men [16,31]. This included an emphasis on fitness and reducing consumption of alcohol, sugar-sweetened beverages, fast foods and processed meals, and foods high in fat content. The website also provided links to publicly available videos and apps for physical activity, with recommendations for free apps to facilitate self-monitoring of dietary intake, weight, and physical activity. To reinforce EPPM messaging across the 12-weeks, participants received weekly text messages that included EPPM constructs. See Fig. 2.

### 2.5 Measures

Demographic information was self-reported by participants via an online questionnaire.

#### 2.5.1 Satisfaction

Overall satisfaction with the program was assessed via self-report at 12-weeks. Two items are reported on a 7-point Likert scale (1 = Very Dissatisfied, 7 = Very Satisfied). “How satisfied were you with the overall ACTIVATE program.” “How satisfied were you with what you achieved in the ACTIVATE program.”.

#### 2.5.2 Weight/Height

Weight was collected via video using a study-issued Bluetooth scale (Renpho). Participants refrained from eating or drinking, except water, for 8 hours and wore light gym clothes and a t-shirt without shoes, socks, and jewelry. Height was assessed via self-report but was not measured directly.

#### 2.5.3 Perceived Risk

The Risk Behavior Diagnosis Scale (RBDS) is 12-item scale that was designed to measure constructs based in the EPPM. The RBDS has demonstrated good internal consistency and predictive validity [32]. Cardiovascular disease was used as the defined health threat and weight loss as the defined recommended response to the health threat. For example, “It is likely that I will get cardiovascular disease if I do not lose weight.”. Participants rated each statement on a 5-point scale (1=Strongly disagree, 5=Strongly agree).

### 2.6 Statistical Analyses

All outcome analyses adhered to the Intent-to-Treat approach, using multiple imputation with 5 imputed datasets for missing cases (n = 5) at follow-up. Data were normally distributed. *T*-tests were used to assess between group differences (intervention *vs.* control) in percent weight change from 0- to 12-weeks and change in EPPM constructs at 2- and 12-weeks. Parallel analyses were used to examine the association between percent weight loss and changes in EPPM constructs. Chi-square analyses were conducted to compare between group differences in the proportion of participants achieving a clinically significant weight loss (>3%) [33]. An alpha level of 0.05 was used. All analyses were conducted using (JMP 15.0, SAS, Inc.; Cary, NC, USA).

## 3. Results

**Fig. 3.**
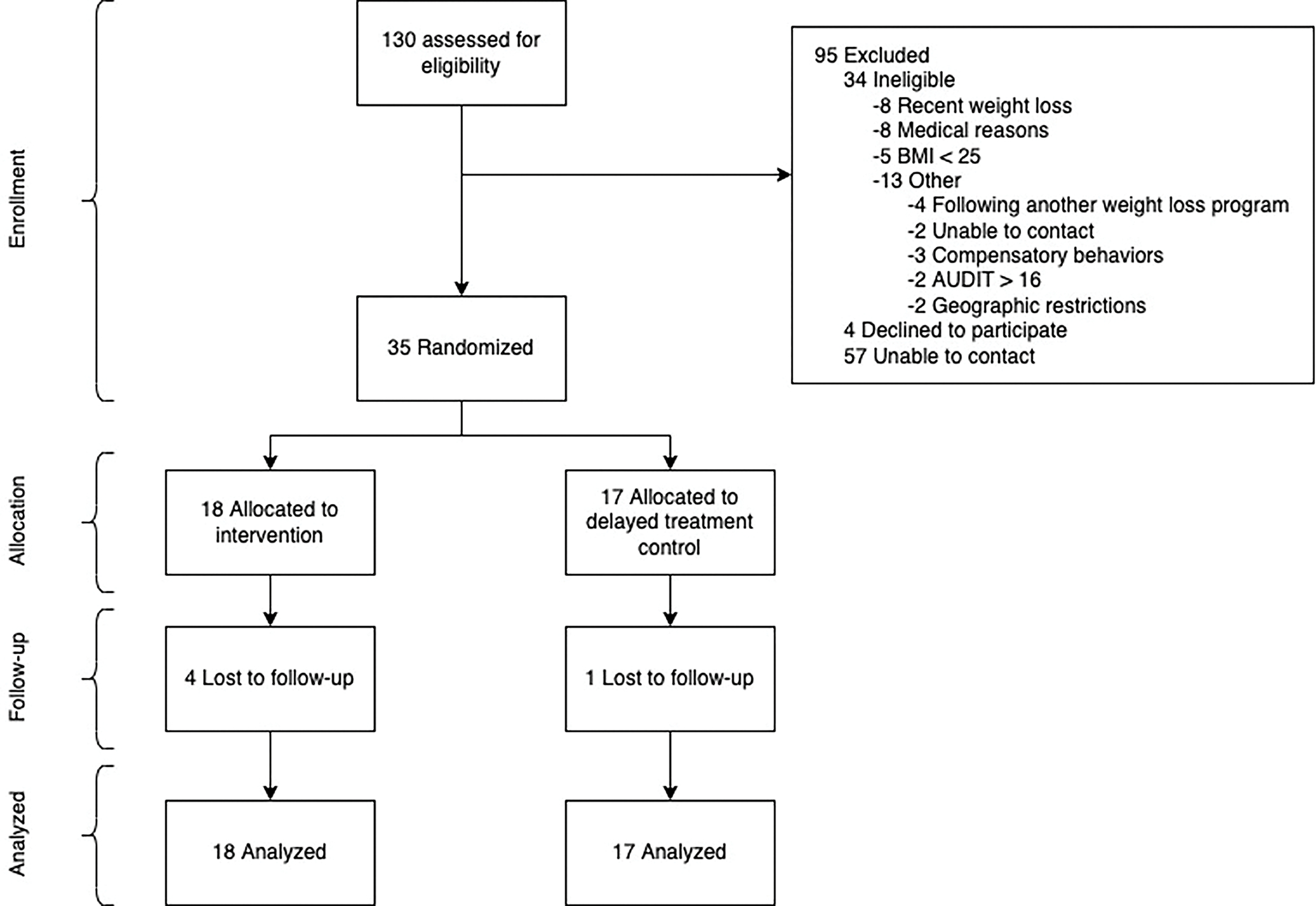
CONSORT flow diagram.

Detailed demographics are displayed overall and by arm in Table 1. The mean age was 29.3 ± 4.27, with an average BMI of 30.8 ± 4.26, and 34% of participants identifying as a racial or ethnic minority. Recruitment and enrollment occurred in a 2-month period. Retention was 86% at 12-weeks, with no statistically significant differences by arm (*p* = 0.17; see Fig. 3 for participant CONSORT flow diagram). Attendance at the virtual group kick-off session was 83%; the 3 participants who missed the live session received a pre-recorded video that covered the same concepts. Overall satisfaction with the ACTIVATE program was 4.2 ± 1.1 on a 7-point scale. Participants overall satisfaction with results they achieved in the intervention was 3.9 ± 0.83 on a 7-point scale.

### 3.1 Weight Change

Participants in the ACTIVATE intervention arm manifested larger reductions in weight compared to the control group (−1.6% ± 2.5 *vs.* +0.31% ± 2.8, *p* = 0.04). See Table 2.

### 3.2 Perceived Risk

Changes in EPPM constructs (response efficacy, self-efficacy, perceived severity, perceived susceptibility) were not associated with percent weight change at 12-weeks (all *p*’s > 0.05) for the intervention or control group. There were no statistically significant differences between participants in the ACTIVATE intervention and control group in changes to EPPM constructs at 12-weeks (all *p*’s >0.05). See Table 2.

## 4. Discussion

The present study sought to determine the feasibility and preliminary efficacy of a self-guided intervention that integrates male-targeted health risk messages on weight loss among young men. Data indicate that participants were moderately satisfied with the intervention and experienced modest weight losses during the 12-week intervention. These findings are consistent with another recent trial that targeted young men in Australia, wherein modest weight loss differences were found between the intervention and control group (−1.3% *vs.* +0.6%) [34]. The prior study was also 12-weeks in duration but was more intensive compared to the current intervention thus supporting the notion that young men may not need high-levels of intensive support in a lifestyle intervention. Instead, an intervention that can potentially be used at their own discretion to meet the demands of this developmental period and preferences of men.

Notably, this trial exceeded recruitment and enrollment goals within the short time span of two months. Remote implementation may have mitigated some of the common barriers reported among young men (time and convenience) [16] that otherwise would have occurred with in-person assessments. Given the notorious challenges recruiting young men in lifestyle interventions [35], these enrollment data are particularly encouraging and suggest this type of male-targeted self-guided program, with remote delivery, might be a promising approach for reaching young men. Furthermore, how the intervention is advertised to young men might be central to engaging this population in weight management. This intervention was designed to meet the specific needs and preferences of young men [16] and used recruitment materials to include various images of young men and messaging (e.g., health risk messages, no health risk message). Though, these recruitment strategies were not experimentally manipulated in this study and varied by outlet. As such, we cannot know which elements of the recruitment ads might have been most effective. Indeed, future studies should consider testing different recruitment strategies that might be effective at engaging this hard-to-reach population.

Lastly, significant changes in EPPM constructs were not observed in the current study. Due to ceiling effects and low variability of these constructs, it is challenging to interpret the association between these constructs with weight change. A possibility for the high perceived risk scores observed at baseline could be due to bias in this treatment-seeking sample wherein the young men who enrolled had greater concern about their weight than young men in the general population. This could also reflect the need for increased dose or tailoring EPPM messaging to enhance these constructs in young men. More research is needed to understand if health risk messaging might be better suited for recruitment materials and whether or not EPPM constructs moderate weight change. One study conducted with Iranian soldiers found that EPPM constructs increased after men received educational training about obesity and weight management, but weight was not assessed as an outcome [25]. Given these findings, along with the challenges of recruiting men into lifestyle interventions, it is worth examining different elements of recruitment materials that might bolster enrollment in this population.

Findings should be interpreted in light of several limitations. First, our sample size was small. Thus, caution should be taken when interpreting and generalizing these results. Additionally, the intervention period was brief and there was only a post-treatment assessment. Therefore, there is a need for replicating these findings in a larger sample over a longer follow-up period. Although 34% of the sample self-identified as racial/ethnic minority, there were no Black men enrolled—even with some advertisements that included image of a young Black man. This underscores the need to improve reach among this population and that superficial adaptations to ads are insufficient. This sample was also highly educated, which coupled with limited racial and ethnic minority enrollment, raises concerns about generalizability. Third, due to the global COVID-19 pandemic, this study was limited to one measure of adiposity because a remote protocol was implemented to minimize risk to participants and study team members. This study would have benefited from collecting body composition and waist circumference, which was originally proposed. As a result, the full cardiometabolic health benefits of this type of self-guided intervention remain unknown. Given the self-guided nature of the program without behavioral targets, combined with the global pandemic and goal of keeping the assessment battery minimal, no self-reported or objective behavioral adherence data were collected. Finally, EPPM messaging was used in recruitment materials, which may have improved enrollment, but might have also increased these constructs prior to the intervention period and contributed to ceiling effects, thereby limiting our ability to differentially promote change in these constructs through this low touch intervention relative to the control arm.

## 5. Conclusions

Our data provides evidence that a brief, self-guided intervention can produce modest weight loss and prevent weight gain among young men. Given the rate of accruing men over a short period of time (2 months) relative to previous work with young adults, these pilot findings suggest that recruiting nationally with a remote protocol is feasible for recruiting this hard-to-reach population. Moreover, the combination of a male-targeted program and health risk messaging shows promise for promoting weight management in this population. However, more research is needed to better understand the specific elements that engage men to lose weight—for incorporation into both recruitment and intervention materials. Overall, these findings highlight that self-guided lifestyle interventions might be a useful, low-cost, and scalable approach for promoting initial weight loss among young men. More testing is needed to better understand which evidence-based components should be added to produce clinically meaningful weight loss among young men, while at the same time, retaining a self-guided approach.

## Figures and Tables

**Table 1.** Participant characteristics (%[n] for categorical variables and mean ± SD for continuous variables).

	Full sample (N = 35)	Treatment (n = 18)	Control (n = 17)

Age	29.6 ± 4.3	29.6 ± 3.8	29.0 ± 4.8
Race/Ethnicity			
American Indian/Alaskan Native	5.9(2)	5.6(1)	6.3(1)
Asian	11.8(4)	11.1(2)	12.5 (2)
Black	0	0	0
Native Hawaiian/Pacific Islander	0	0	0
Non-Hispanic White	65.7 (23)	72.2(13)	58.8(10)
Other	8.8 (3)	5.6(1)	12.5 (2)
Latino	21.2 (7)	22.2 (4)	20.0 (3)
Multiracial	14.7(5)	16.7(3)	12.5 (2)
Work/School status			
Working full-time	73.3 (22)	78.6(11)	68.8(11)
Working part-time	13.3 (4)	14.3 (2)	12.5 (2)
Student full-time	16.7(5)	14.3 (2)	18.8 (3)
Student part-time	0	0	0
Hours worked weekly	39.9 ± 9.5	41.3 ± 10.5	38.5 ± 8.6
Level ofeducation			
Some college	13.3 (4)	14.3 (2)	12.5 (2)
College graduate	46.7(14)	57.1 (8)	37.5 (6)
Postgraduate degree	40.0(12)	13.3 (4)	50.0 (8)
Relationship status			
Married	48.3(14)	46.2 (6)	50.0 (8)
Single	34.5(10)	46.2 (6)	25.0 (4)
Living with partner	17.2(5)	7.7(1)	25.0 (4)
Baseline outcomes			
Baseline BMI	30.8 ± 4.2	30.9 ± 4.7	30.7 ± 3.9
Baseline Weight (kg)	96.8 ± 15.5	98.3 ± 18.1	95.1 ± 14.2
Response efficacy	4.6 ± 0.7	4.7 ± 0.5	4.6 ± 0.8
Self-efficacy	3.7 ± 0.8	3.7 ± 0.8	3.7 ± 0.8
Perceived severity	4.7 ± 0.5	4.7 ± 0.4	4.7 ± 0.5
Perceived susceptibility	3.7 ± 1.0	3.9 ± 0.9	3.5 ± 1.1

**Table 2.** Change in outcomes by arm (mean ± SD [95% Confidence Interval]).

	Treatment	Control	*p*-value	Cohen’s d

Weight change (%)	−1.6% ± 2.5 [−2.8,−0.36]	0.31% ± 2.8 [−1.3, 1.7]	0.042	0.72
2-week perceived risk change				
Perceived severity	0.13 ± 0.38 [−0.06, 0.31]	0.05 ± 0.35 [−0.13, 0.23]	0.551	0.16
Perceived susceptibility	−0.04 ± 0.58 [−0.33, 0.25]	−0.05 ± 0.70 [−0.42, 0.30]	0.921	0.04
Response efficacy	0.07 ± 0.59 [−0.22, 0.36]	−0.01 ± 0.38 [−0.22, 0.18]	0.583	0.19
Self-efficacy	0.22 ± 0.85 [0.20, 0.64]	0.11 ± 0.59 [−0.19,0.41]	0.661	0.22
12-week perceived risk change				
Perceived severity	0.08 ± 0.41 [−0.12, 0.28]	−0.18 ± 1.4 [−0.90, 0.54]	0.640	0.64
Perceived susceptibility	−0.10 ± 1.1 [−0.67, 0.45]	0.02 ± 1.1 [−0.56,0.60]	0.317	0.32
Response efficacy	0.05 ± 0.44 [−0.17, 0.27]	0.02 ± 0.36 [−0.16, 0.21]	0.829	0.07
Self-efficacy	0.19 ± 0.59 [−0.11,0.48]	0.13 ± 0.73 [−0.24, 0.50]	0.810	0.13

## Abbreviations

BMI: body mass index
EPPM: extended parallel process model
RBD: risk behavior diagnosis
